# Prevalence of Clinical Signs and Symptoms of Temporomandibular Joint Disorders Registered in the EUROTMJ Database: A Prospective Study in a Portuguese Center

**DOI:** 10.3390/jcm12103553

**Published:** 2023-05-18

**Authors:** David Faustino Ângelo, Beatriz Mota, Ricardo São João, David Sanz, Henrique José Cardoso

**Affiliations:** 1 Instituto Português da Face, 1050-227 Lisboa, Portugal; 2 Faculdade de Medicina, Universidade de Lisboa, 1649-028 Lisboa, Portugal; 3 Serviço de Estomatologia, Centro Hospitalar Universitário Lisboa Norte (CHULN), 1649-035 Lisboa, Portugal; 4 Department of Computer Science and Quantitative Methods, School of Management and Technology, Polytechnic Institute of Santarém, 2001-904 Santarém, Portugal; 5 CEAUL—Centro de Estatística e Aplicações, Faculdade de Ciências, Universidade de Lisboa, 1749-016 Lisboa, Portugal

**Keywords:** temporomandibular joint disorders, parafunctional habits, risk factors, chronic diseases

## Abstract

Temporomandibular joint disorders (TMDs) are characterized by their multifactorial etiology and pathogenesis. A 3-year prospective study was conducted in a Portuguese TMDs department to study the prevalence of different TMDs signs and symptoms and their association with risk factors and comorbidities. Five hundred ninety-five patients were included using an online database: EUROTMJ. Most patients were female (80.50%), with a mean age of 38.20 ± 15.73 years. The main complaints were: (1) temporomandibular joint (TMJ) clicking (13.26%); (2) TMJ pain (12.49%); (3) masticatory muscle tension (12.15%). The main clinical findings were myalgia (74%), TMJ clicking (60–62%), and TMJ arthralgia (31–36%). Risk factors such as clenching (60%) and bruxism (30%) were positively associated with TMJ pain and myalgia. Orthodontic treatment (20%) and wisdom tooth removal (19%) were positively associated with TMJ clicking, while jaw trauma (6%), tracheal intubation (4%) and orthognathic surgery (1%) were positively associated with TMJ crepitus, limited mandibular range of motion, and TMJ pain, respectively. In total, 42.88% of TMDs patients had other associated chronic diseases, most of them were mental behavioral or neurodevelopmental disorders (33.76%), namely, anxiety (20%) and depression (13%). The authors also observed a positive association of mental disorders with the degree of TMJ pain and myalgia. The online database seems to be a relevant scientific instrument for healthcare providers who treat TMDs. The authors expect that the EUROTMJ database can serve as a milestone for other TMDs departments.

## 1. Introduction

Temporomandibular joint disorders (TMDs) are a group of dysfunctions that appear to be of multifactorial origin, affecting the temporomandibular joint (TMJ), masticatory muscles, and adjacent structures [1,2]. Nowadays, TMDs are considered the most frequent cause of chronic orofacial pain of non-dental origin and the third stomatological disorder leading to pain and disability. In addition, myofascial TMDs are documented as the most frequent subtype, followed by internal derangements such as disc displacement and TMJ arthralgia [3,4].

It is estimated that TMDs affect about 31% of the adult population and 11% of children/adolescents [5]. TMDs prevalence is significantly higher in women (female/male ratio 5:1) and younger subjects [6]. However, TMD signs seem to increase with age [1,2,7].

Psychosocial, environmental, biological, and neurophysiological factors are considered etiological entities significantly associated with TMD symptoms. Factors such as emotional stress (anxiety and depression), bruxism, occlusal disharmony, orthodontic treatment, masticatory dysfunction, and postural deviation are reported to increase the risk for TMDs [1,4,7].

The most prominent symptoms are restricted joint function with alteration of the mandibular physiological dynamics, muscular or articular pain that intensifies with mastication, headache, and TMJ noises. Limited mandibular range of motion, pain, crepitation, or clicking in TMJ palpation are all common signs of TMDs assessed during a clinical examination [1,2].

This study attempts to evaluate and analyze the prevalence of TMD clinical signs and symptoms in a Portuguese TMD department and their association with various sociodemographic and individual factors such as age, gender, oral behaviors, risk factors, and other comorbidities.

## 2. Materials and Methods

### 2.1. Database Description

**Figure 1:**
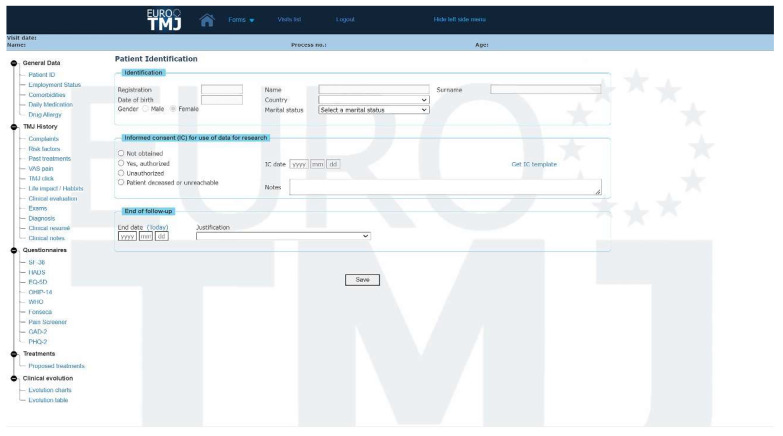
EUROTMJ database screen.

European temporomandibular joint (EUROTMJ) is an online electronic medical database that allows data collection from patients with TMDs (Figure 1). The patient’s data is encrypted and only accessible through a password attributed to the clinician. The database works in the English language. EUROTMJ displays a tree format table of contents (Figure 1). The database is constituted with different menus: general data (patient ID, employment status, comorbidities, daily medication, drug allergy); TMJ history (complaints, risk factors, past treatments, VAS Pain, TMJ click, life impact/habits; clinical evaluation; exams, diagnosis, clinical resume, notes); questionnaires (SF-36, HADS, EQ-5D, OHIP-14, WHO, Fonseca, Pain Screener, GAD-2, PHQ-2); treatments (Proposed treatments); and clinical evaluation (evolution charts, evolution table).

### 2.2. Study Design and Data Collection

A 3-year prospective study was conducted in a Portuguese TMD department from 1 August 2019 to 1 August 2022. This study was approved by the Instituto Português da Face ethics committee (PT/IPFace//RCT/0822/01). All enrolled patients gave their informed consent in writing, following current legislation. The inclusion criteria were: (1) registration of all the variables under study in a first consultation and (2) clinical diagnosis of TMDs. The exclusion criteria were severe medical problems or impaired cognitive capacity. All patients with inclusion criteria over these three years were included. Descriptive data and clinical outcomes were registered in EUROTMJ. Demographic data (date of birth and gender) was recorded. In the first consultation, patients were instructed to answer questions regarding their complaints (TMJ pain, TMJ clicking, TMJ crepitus, limited mouth opening, masticatory muscles tension, cervical muscles tension, tinnitus, TMJ edema, and vertigo), laterality (when applicable), and duration of the symptoms (<3M; 3M–1Y; 1Y–5Y > 5Y). They were also asked about parafunctional habits in their daily life, such as bruxism and clenching. A clinical record registered other comorbidities and potential risk factors for TMDs. The TMJ pain recording was accessed in the right and left joints through the Visual Analog Scale (VAS, 0–10, with 0 being no pain; 1–3: mild, 4–6: moderate, and 7–10 severe pain). The VASLIFE was accessed by asking: “If you could give a life impact score to your TMJ problem on a 0 to 10 scale, where 0 means no impact, and 10 means the maximum impact possible, what would be your score?” [8]. In clinical evaluation, maximum mouth opening (MMO, mm), the presence of clicks, crepitus, arthralgia (right and left joint), and myalgia were recorded. MMO was accessed using a certified ruler between the incisor teeth (TheraBite Jaw ROM Scale). Limited mouth opening was registered when MMO < 40 mm [9,10]. Myalgia was diagnosed according to a positive clinical history for: (1) in the past 30 days, pain in the jaw, in front, or directly in the ear, with confirmation of pain through palpation of the masticatory muscles by the examiner and (2) accompanied pain with jaw movement, function or parafunction, and a positive clinical evaluation for palpation pressure (5 s/1 kg pressure) in masseter and temporalis muscles as defined in DC/TMD [9,10]. Myalgia was graded accordingly with pain intensity in each muscle: 0 = no pain/pressure only; 1 = mild pain; 2 = moderate pain; 3 = severe pain [11]. Arthralgia was reported if verified: (1) history of pain in the TMJ area and (2) accompanied pain with jaw movement, function, or parafunction [9,10]. The level of TMJ arthralgia was registered through the pain on palpation of the lateral pole or around or pain on maximum unassisted or assisted opening, lateral, or protrusive movements. The same clinician (D.F.A.) performed the clinical evaluation for all patients.

### 2.3. Statistical Analysis

Data were analyzed using the GraphPad Prism (v9, (Boston, MA, USA) and IBM SPSS (v26, Armonk, NY, USA) software. The variables were expressed as the mean (±standard deviation (SD)) or frequency (%). The biserial correlation Pearson Test (rpb) assessed the variables’ correlation. The non-parametric Chi-square test (χ^2^) was used to determine the associations’ presence, and its intensity was measured using Cramér’s V Coefficient (φc). A *p*-value < 0.05 was considered statistically significant. For a graphic representation of risk factors and other comorbidities, a percentage >1% was assumed.

## 3. Results

A total of 595 patients were registered in the EUROTMJ database. The mean age was 38.20 ± 15.73 years at the first visit, 479 (80.50%) of whom were female gender (Table 1). 86.39% of the patients presented symptoms bilaterally, while 6.89% and 6.72% presented only on the right and left, respectively.

**Figure 2:**
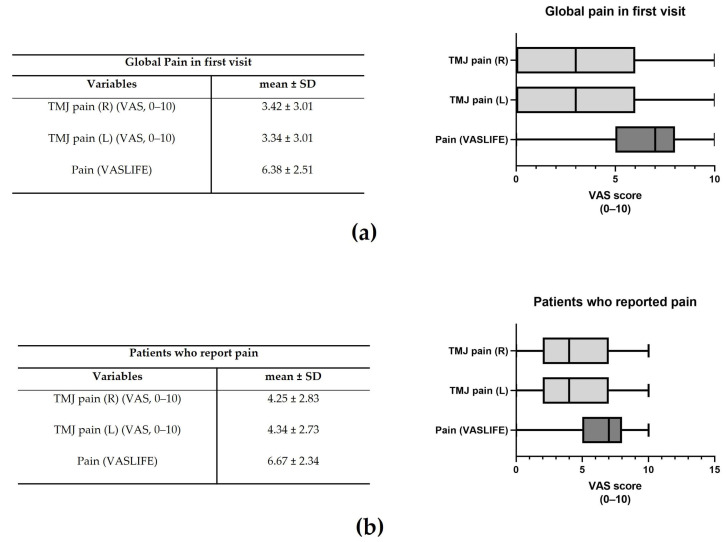
Global pain in the first visit (**a**) and Visual Analogue Score (VAS) in the patients that reported pain (**b**). The representative median VAS values in the range [Q1 − 1.5IQR; Q3 + 1.5IQR] are shown in the right graphs where IQR = interquartile range and Q1 and Q3 are, respectively, the 1st and 3rd quartiles (*n* = 595).

The mean global TMJ pain in the right and left joints was 3.42 ± 3.01 and 3.34 ± 3.01, respectively (Figure 2a). The impact on life (VASLIFE) was 6.38 ± 2.51 (Figure 2a).

**Figure 3:**
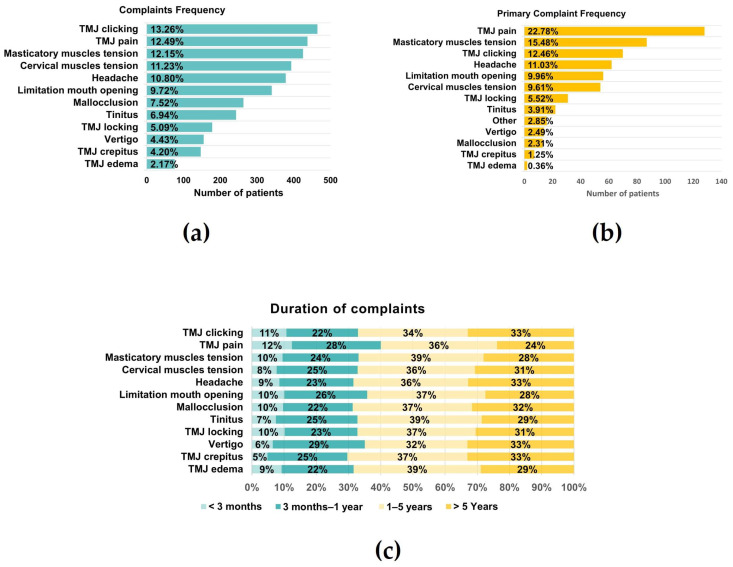
Global Complaints. (**a**) Complaints frequency; (**b**) Primary complaint frequency; (**c**) Duration of each complaint (*n* = 595).

Considering only those patients who presented pain, pain right and left was 4.25 ± 2.83 and 4.34 ± 2.73, whereas VASLIFE was 6.67 ± 2.34 (Figure 2b). The main complaints of the patients were TMJ clicking (13.26%), TMJ pain (12.49%), and masticatory muscle tension (12.15%), while the least frequent were vertigo (4.43%), crepitus (4.20%), and edema (2.17%) (Figure 3a). However, considering the patient’s main complaint, the one which presented the higher predominance was TMJ pain (22.78%) (Figure 3b). There was also a high prevalence of symptom duration between 1–5 years (32–39%) and over 5 years (24–33%), while a low frequency of symptoms under 3 months was verified (5–12%) (Figure 3c).

**Figure 4:**
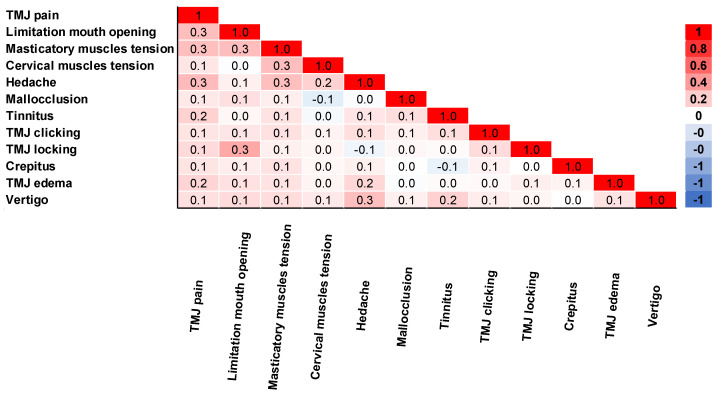
Correlation matrix of complaints obtained by biserial correlation Pearson Test (*n* = 595).

The correlation of complaints was analyzed in Figure 4. TMJ pain was moderately correlated with limited mouth opening, masticatory muscle tension, and headache (rpb = 0.3). TMJ pain also had a small correlation with other complaints (rpb = 0.1–0.2). Limitation of mouth opening was moderately correlated with TMJ locking and masticatory muscle tension (rpb = 0.3). Masticatory muscle tension was moderately correlated with headache and cervical muscle tension (rpb = 0.3). Additionally, headache was a moderate correlation with vertigo (rpb = 0.3).

Regarding parafunctional habits, 180 (30%) of the patients reported positively to bruxism, 138 (23%) at night, 35 (6%) at day and night, and 7 (1%) during the day. On the contrary, 212 (36%) said that they do not have bruxism habits, and 203 (34%) do not know (Table 2). Regarding clenching, 359 (60%) of the patients answered that they do it, while 159 (27%) did not, and 77 (13%) did not know.

**Figure 5:**
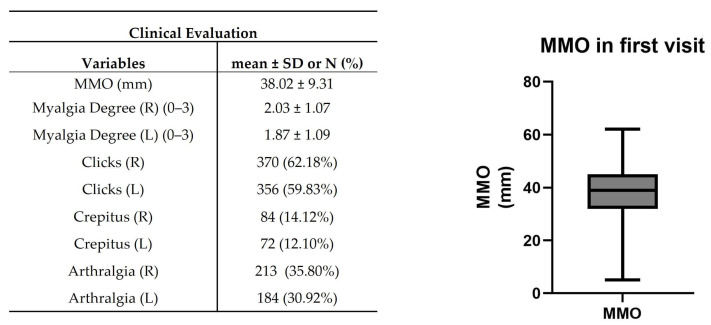
Clinical evaluation in the first visit. Maximum mouth opening (MMO), muscle tenderness, clicks, crepitus, and arthralgia were registered. The right graph shows the representative median MMO value in the range [Q1 − 1.5IQR; Q3 + 1.5IQR] where IQR = interquartile range and Q1 and Q3 are, respectively, the 1st and 3rd quartiles (*n* = 595).

In clinical evaluation, patients presented 38.02 ± 9.31 mm of MMO (Figure 5). The mean myalgia degree was 2.03 ± 1.07 and 1.87 ± 1.09 on the right and left sides, respectively. A total of 370 (~62%) and 356 (~60%) patients presented clicks in the right and left TMJ, while 84 (~14%) and 72 (~12%) patients were verified crepitus (Figure 5). A suggestive pain indicative of TMJ arthralgia was detected in 213 (~36%) and 184 (~31%) patients in the right and left TMJ (Figure 5).

**Figure 6:**
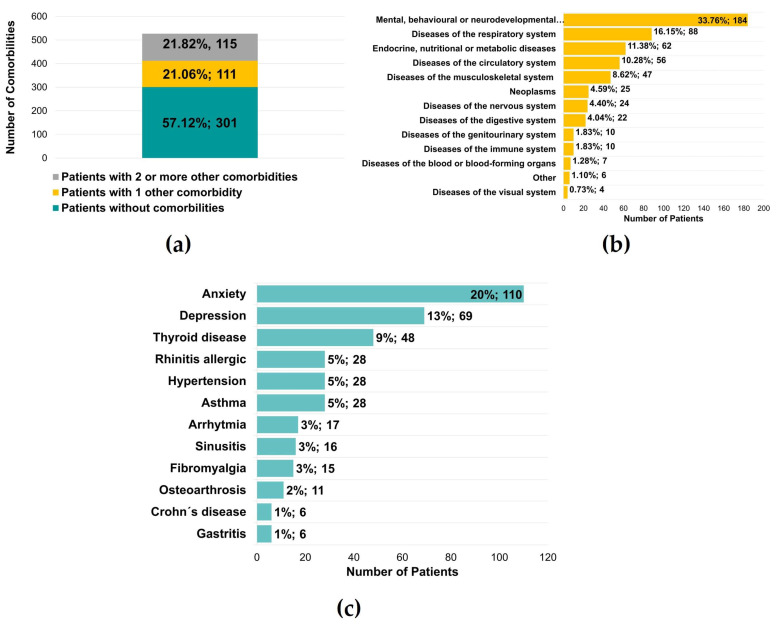
Other comorbidities identified in patients with temporomandibular disorders. (**a**) Number of patients without or with at least 1 or 2 comorbidities (*n* = 527) (**b**) Number of patients for a group of comorbidities (*n* = 545) (**c**) Number of patients with specific comorbidities (*n* = 545).

Five hundred twenty-seven patients were also questioned regarding the diagnosis of other diseases, and it was found that 226 (42.88%) of the patients had another illness, with 111 (21.06%) with one condition and 115 (21.82%) with two or more diseases (Figure 6a). In a total of 545 reported medical conditions, there was a significant predominance of mental, behavioral, or neurodevelopmental disorders (184 patients, 33.76%) (Figure 6b). These values were supported by the diagnosis of anxiety and depression, verified in 110 (20%) and 69 (13%) patients, respectively (Figure 6c). The authors observed an essential percentage of patients with respiratory and endocrine/metabolic diseases, namely thyroid pathology (48 patients, 9%) (Figure 6c).

**Figure 7:**
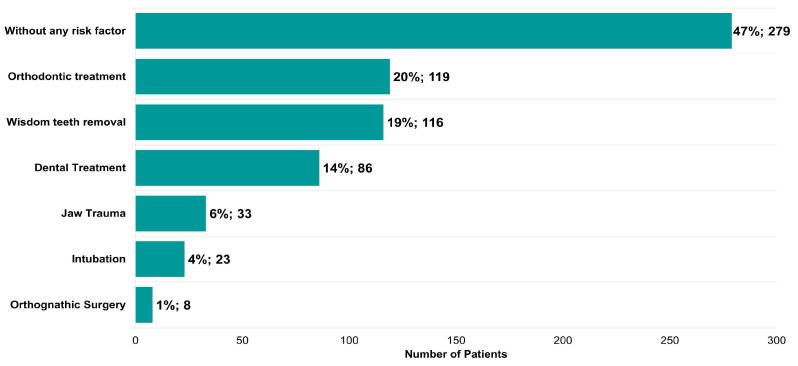
Risk factors identified in patients with symptoms of temporomandibular disease (*n* = 595).

Potential risk factors described in the literature for triggering the onset of TMDs were also identified (Figure 7). In total, 53% of the patients presented at least one risk factor (Figure 7). Orthodontic treatment (119, 20%), wisdom tooth removal (116, 19%), dental treatment (86, 14%), jaw trauma (33, 6%), intubation (23, 4%), and orthognathic surgery (8, 1%) represent a large proportion of the risk factors identified (Figure 7).

The association of clinical variables and TMJ pain with demographic data, parafunctional habits, risk factors, and other comorbidities were then studied (Table S1).

In the demographic data, sex has a strong association with pain (*p* < 0.001, φc = 0.189, Table 3) and limitation of mouth opening (*p* < 0.001, φc = 0.154, Table 3), and a very strong association with the degree of myalgia (*p* < 0.001, φc = 0.277, Table 3).

**Figure 8:**
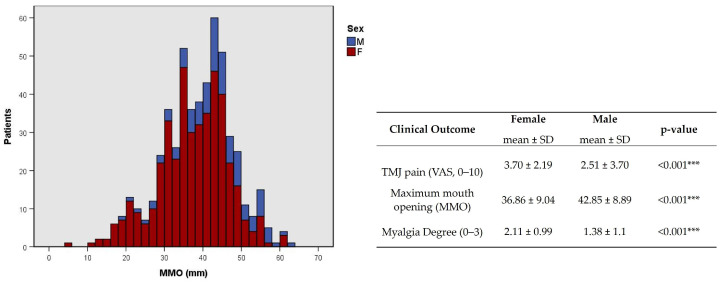
Distribution of maximum mouth opening (MMO) and statistical difference between TMJ pain, MMO, and myalgia degree by sex. F-female; M-male. *** *p* < 0.001.

This association was corroborated by a higher degree of TMJ pain and myalgia and a lower MMO average in females (*p* < 0.001, Figure 8). At younger ages, the presence of clicks was more common (*p* = 0.002, φc = 0.177, Table 3), while at older ages, the presence of crepitus was more common (*p* < 0.001, φc = 0.229, Table 3).

Parafunctional habits have also been shown to have an association with the clinical variables: clenching was strongly associated with myalgia degree (*p* = 0.008, φc = 0.159, Table 4); bruxism was strongly and moderately associated with myalgia degree and TMJ pain (*p* = 0.006, φc = 0.186 and *p* = 0.035, φc = 0.148, Table 4).

Evaluating the association of risk factors with clinical variables revealed: past intubation was weakly related to limited mouth opening (*p* = 0.047, φc = 0.082, Table 5), while past orthognathic surgery showed a moderate association with TMJ pain (*p* = 0.011, φc = 0.137, Table 5); orthodontic treatment and wisdom teeth removal were weakly associated with TMJ clicks (*p* = 0.026, φc = 0.094; *p* = 0.038, φc = 0.088, Table 5) and jaw trauma with TMJ crepitus (*p* = 0.037, φc = 0.093, Table 5).

The presence of other comorbidities has been shown to have a strong association with the degree of TMJ pain and myalgia (*p* = 0.002, φc = 0.131 and *p* < 0.001, φc = 0.185, Table 6). Within the identified disease classes, mental, behavioral, or neurodevelopmental disorders were strongly associated with the degree of myalgia and a moderate association with TMJ pain intensity (*p* < 0.001, φc = 0.202 and *p* = 0.008, φc = 0.142, Table 6). Circulatory system diseases were moderately associated with the absence of TMJ clicks (*p* = 0.003, φc = 0.125, Table 6).

## 4. Discussion

TMDs are one of the principal causes of chronic facial pain, affecting a considerable part of the population [12]. This 3-year prospective study provides a comprehensive and detailed characterization of the TMDs population in this research and demonstrates possible correlations with other comorbidities or risk factors. In this study, 80% of our patients were females with a mean age of 38 years. The age pattern and proportion of females were consistent with the results of other studies [3,13,14]. Iodice, et al. [3] revealed that this phenomenon might result from the biological, behavioral, psychological, and/or social factors associated with the female gender [3].

In our current study, the most common TMDs symptom was TMJ clicking (13.26%), as reported in other studies [3,15,16], followed by TMJ pain (12.49%) and masticatory muscle tension (12.15%). However, considering only the patient’s main complaint, we found that 22.78% referred to TMJ pain as their leading symptom. Additionally, in our study, the duration of complaints was mainly 1–5 and over 5 years, with a low occurrence of symptoms under 3 months. Therefore, we hypothesize that most patients take some time to associate the symptoms with TMDs and find adequate medical opinion. This correlates with the fact that overall, the frequency of seeking treatment increases as the symptoms interfere with day-to-day activities. In addition, we also observed that TMDs pain occurs as part of a group of symptoms rather than as a single entity [3].

In our study, TMDs signs are more frequent than symptoms [2,17]. This can partly be explained by the difference between the prevalence of TMJ clicking as a complaint (13.26%) or clinical signs during the medical evaluation. Despite being a TMDs symptom, TMJ clicking can also be a sign of TMDs. Nevertheless, the most common TMDs sign was TMJ sounds (~62% and ~60% clicking in the right and left TMJ; ~14% and ~12 crepitus in the right and left TMJ). Suggestive pain is indicative of possible TMJ arthralgia (~36% and ~31% in the right and left TMJ), masticatory muscles myalgia (2.03 ± 1.07 and 1.87 ± 1.09 on the right and left sides, respectively), and MMO (38 ± 9.31 mm) was also evaluated. This data suggests that an expert clinical evaluation can sometimes valorize signs that patients are unaware of. Previous studies correlate the association between TMDs signs and symptoms with age, gender, and TMDs diagnosis. TMJ clicking has been shown to be common in younger subjects, while TMJ crepitation showed a stronger association with higher age [3,18]. This result is in accordance with our study, as our population was mainly younger subjects and the predominant clinical sign was TMJ clicking. At the same time, crepitus was more prevalent in older patients. This result is in agreement that the presence of crepitus is related to the diagnosis of osteoarthritis, which is more common in older patients [19]. Additionally, TMJ pain and myalgia were significantly higher in females, while MMO was significantly lower. This result shows that besides the prevalence of the disease in women, the symptoms are also more exacerbated. The correlation between signs and symptoms can contribute to an appropriate TMDs diagnosis. Previous studies demonstrate that muscle pain and tenderness may indicate myofascial disorders, while TMJ pain on palpation and limited mouth opening (LMO) can suggest intra-articular conditions. In our research, this was observed in pain indicative of arthralgia (~36% and ~31% in the right and left TMJ) and a mean MMO of 38 ± 9.31 mm, encountering a limitation of mouth opening as a cut-off of MMO < 40 mm was defined in our study.

Furthermore, TMJ pain was moderately correlated with LMO, while masticatory muscle tension had a moderate correlation with headache and cervical muscle tension. Almoznino, et al. [20] also showed higher cervical tenderness scores in myogenous TMDs patients and a positive association with TMJ pain and headaches. This connection can be related to myofascial trigger points in the trapezius that potentiate electromyographic changes in the masticatory muscles [21]. LMO was moderately associated with TMJ locking. On the other hand, TMJ clicking is considered a potential sign of disc displacement with reduction. Otherwise, LMO can be a typical sign of disc displacement without reduction [2]. In the present study, ~62% and ~60% of the patients presented TMJ clicking in the right and left TMJ, respectively, and the mean of MMO measured was 38 ± 9.31 mm. By analyzing these results, we observed that over one-half of the patients evaluated had signs of disc displacement with reduction. However, on average, the majority of the patients presented LMO. Previous studies support these findings as they linked LMO with other entities besides disc displacement without reduction, such as myalgia and degenerative joint disease associated with the normal aging process, possibly explaining the high percentage of individuals with LMO in this study [16]. In addition, some authors described chronic disc displacement without reduction with the absence of LMO, while acute disease with the presence of LMO [22,23].

The most frequent possible causes for TMDs described by the patients included: (1) parafunctional habits (clenching (60%), bruxism (30%)); (2) previous orthodontic treatment (20%); (3) wisdom tooth removal (19%), (4) general dental treatment (14%), (5) jaw trauma (6%), (6) tracheal intubation (4%), and (7) orthognathic surgery (1%). Our study shows a strong association between clenching and bruxism with the degree of myalgia and a moderate association between clenching and TMJ pain. In addition, previous tracheal intubation, orthognathic surgery, and wisdom tooth removal were positively associated with a limited mandibular range of motion, TMJ pain, and TMJ clicking, respectively. These findings are in accordance with previous studies, such as Marklund et al. [24], which performed a 2-year prospective observational study and concluded that biomechanical factors, such as bruxism, mandibular instability, and malocclusion, were linked to the incidence and persistence of TMJ signs and symptoms. Moreover, trauma and long-standing load may also contribute to the development and course of TMDs [24,25].

Regarding other comorbidities, it is unclear to which extent TMDs may reflect symptoms or manifestations of underlying diseases. In the current study, although most of the patients evaluated had no comorbidities (57.12%), 42.88% of TMDs had other conditions and 21.06% presented only one disease. In comparison, 21.82% of the patients referred to two or more diseases.

Almost one-third of these patients reported mental behavior and neurodevelopmental diseases, a pertinent factor for many patients with TMDs. A total of 20% had positive results for anxiety and 13% for depression. This study shows a correlation between mental behavioral and neurodevelopmental diseases with TMJ pain and myalgia. This result is consistent with other studies that demonstrated significant associations between stress, anxiety, depression, and TMDs [26,27]. Higher levels of stress and depression are related to changes in electrical potentials and asymmetry of the masticatory muscles during clenching [28,29]. In fact, higher levels of masticatory muscle pain perception were demonstrated in response to psychological stress in Sprague-Dawley rats [30]. Previous and present results strongly support an association and should be aware that specialists to keep psychosocial modulators as possible treatment-associated modalities. In addition, other chronic diseases summarized in Figure 6, such as respiratory and endocrine alterations, might be related to TMDs. This is corroborated in other population studies as there is evidence for an association between impaired general health and TMDs, suggesting that TMDs symptoms may share characteristics with other chronic conditions [31,32].

One of the strengths of this study was the consistency of a questionnaire and clinical examination to obtain reliable results for TMJ signs and symptoms in the first appointment. In addition, the EUROTMJ database allows the collection of data and evaluation of TMDs-related symptoms, correlating the findings with TMDs signs. This can be a valuable and applicable platform for TMDs screening and treatment decisions. Additionally, only one specialist examined all the patients in a TMDs-specialized appointment, making it an advantage of this study. However, as with most studies, the present analysis has limitations. Firstly, the database and questionnaire are not validated; furthermore, information bias might be present since the parameters evaluated using the VAS scale could be over or underestimated by the patients. Additionally, the evaluation of the correlation between TMDs signs and symptoms and TMJ diagnosis and proposed treatment was not included in this study, making it a limitation. Moreover, this study is single-center, which should be reproduced in a multi-center study.

## 5. Conclusions

TMDs are a group of dysfunctions that affect a considerable part of the population nowadays, mainly younger female patients. In the subjects included in this study, TMDs were shown to be a group of disorders with a broad spectrum of clinical manifestations, pathophysiology, and associated comorbid conditions. Significant associations between TMDs signs and symptoms with intrinsic characteristics, such as age, gender, and parafunctional habits, such as clenching and bruxism, have been made. Positive correlations were also made with mental behavior and neurodevelopmental diseases, such as anxiety and depression, and other comorbidities, such as respiratory and endocrine alterations. Interestingly, risk factors such as wisdom tooth removal, orthodontic treatment, jaw trauma, tracheal intubation, and orthognathic surgery may increase the susceptibility to developing TMDs clinical symptoms. However, more studies are needed to understand such associations. We believe this data will serve as a milestone in providing helpful information for researchers and healthcare providers treating patients with TMDs.

## Figures and Tables

**Table 1:** Demographic data and side of the TMJ symptoms.

Variables	*n* (%), or Mean ± SD	
Number of patients	595	
Sex	Female	479 (80.50%)
	Male	116 (19.50%)
Age	38.20 ± 15.73	
Side of the joint with symptoms	Only Right	41 (6.89%)
	Only Left	40 (6.72%)
	Bilateral	514 (86.39%)

**Table 2:** Parafunctional habits. Frequency of Clenching and bruxism habits. *n* = 595.

Parafunctional Habits		
Variables		*n* (%)
Bruxism	Day	7 (1%)
	Night	138 (23%)
	Day and Night	35 (6%)
	No	212 (36%)
	Does not know	203 (34%)
Clenching	Yes	359 (60%)
	No	159 (27%)
	Does not know	77 (13%)

**Table 3:** Association table with demographic data and TMJ pain and clinical variables. Significant values are in bold.

TMJ Pain and Clinical Variables						
Demographic Data	TMJ Pain	Limitation of Mouth Opening	Myalgia Degree	TMJ Clicks	TMJ Crepitus	Arthralgia
	*p*-Value, Cramer’s V					
Sex	**<0.001, 0.189**	**<0.001, 0.154**	**<0.001, 0.277**	0.698, 0.016	0.179, 0.060	0.143, 0.064
Age	0.889, 0.060	0.543, 0.072	0.456, 0.088	**0.002, 0.177**	**<0.001, 0.229**	**0.032, 0.142**

**Table 4:** Association table with parafunctional habits and TMJ pain and clinical variables. Significant values are in bold.

TMJ Pain and Clinical Variables						
Parafunctional Habits	TMJ Pain	Limitation of Mouth Opening	Myalgia Degree	TMJ Clicks	TMJ Crepitus	Arthralgia
	*p*-Value, Cramer’s V					
Clenching	0.203, 0.094	0.340, 0.042	**0.008, 0.159**	0.760, 0.013	0.571, 0.026	0.677, 0.020
Bruxism	**0.035, 0.148**	0.732, 0.017	**0.006, 0.186**	0.890, 0.012	0.496, 0.036	0.364, 0.048

**Table 5:** Association table with risk factors and TMJ pain and clinical variables. Significant values are in bold.

Global Pain and Clinical Variables						
Risk Factors	TMJ Pain	Open Mouth Limitation	Myalgia Degree	TMJ Clicks	TMJ Crepitus	Arthralgia
	*p*-Value, Cramer’s V					
Dental treatment	0.343, 0.075	0.168, 0.057	0.891, 0.035	0.698, 0.016	0.270, 0.049	0.065, 0.080
Orthodontic treatment	0.419, 0.069	0.116, 0.064	0.386, 0.077	**0.026, 0.094**	0.238, 0.053	0.523, 0.028
Intubation	0.489, 0.064	**0.047, 0.082**	0.323, 0.082	0.169, 0.058	0.135, 0.067	0.731, 0.015
Orthognathic surgery	**0.011, 0.137**	0.234, 0.049	0.286, 0.086	0.858, 0.008	0.313, 0.045	0.524, 0.028
Wisdom teeth removal	0.537, 0.060	0.115, 0.065	0.879, 0.036	**0.038, 0.088**	0.824, 0.010	0.729, 0.015
Jaw trauma	0.216, 0.087	0.326, 0.040	0.179, 0.098	0.732, 0.014	**0.037, 0.093**	0.201, 0.056

**Table 6:** Association table of other comorbidities and TMJ pain and clinical variables. Significant values are in bold.

TMJ Pain and Clinical Variables						
Other Comorbidities	TMJ Pain	Limitation of Mouth Opening	Myalgia Degree	TMJ Clicks	TMJ Crepitus	Arthralgia
	*p*-Value, Cramer’s V					
*n* (no, 1, ≥2)	**0.002, 0.131**	0.354, 0.059	**<0.001, 0.185**	0.125, 0.086	0.490, 0.054	0.786, 0.030
Group of other Comorbidities						
Mental, behavioral, or neurodevelopmental disorders	**0.008, 0.142**	0.678, 0.017	**<0.001, 0.202**	0.708, 0.016	0.677, 0.019	0.645, 0.020
Diseases of the respiratory system	0.492, 0.064	0.475, 0.029	0.138, 0.103	0.381, 0.037	0.236, 0.053	0.835, 0.009
Endocrine, nutritional, or metabolic diseases	0.154, 0.094	0.798, 0.011	0.195, 0.096	0.264, 0.047	0.370, 0.040	0.487, 0.030
Diseases of the circulatory system	0.990, 0.014	0.679, 0.017	0.956, 0.025	**0.003, 0.125**	0.066, 0.083	0.416, 0.035
Diseases of the musculoskeletal system	0.228, 0.085	0.584, 0.022	0.719, 0.051	0.979, 0.001	0.125, 0.069	0.253, 0.050

## Data Availability

Not applicable.

## Supplementary Materials

The following supporting information can be downloaded at: https://www.mdpi.com/article/10.3390/jcm12103553/s1, Table S1: Relative frequency (%) of the TMJ pain and clinical variables.
