# Supplementary material for: Prevalence of Clinical Signs and Symptoms of Temporomandibular Joint Disorders Registered in the EUROTMJ Database: A Prospective Study in a Portuguese Center
Source: J Clin Med. 2023 May 18;12(10):3553. doi: 10.3390/jcm12103553 (PMC10219561; doi:10.3390/jcm12103553)
Supplement: Supplementary file 1 [file jcm-12-03553-s001.zip › jcm-2410091-supplementary.pdf]

Table S1. Relative frequency (%) of the TMJ pain and clinical variables (open mouth limitation, clicks, crepitus and arthralgia) with demographic data, parafunctional habits, risk factors and other comorbidities. F – female; Male. Significant values are in bold.

	TMJ Pain				Open Mouth Limitation		Myalgia Degree				TMJ Clicks		TMJ Crepitus		Arthralgia	
	No	Mild	Moderate	Severe	No	Yes	0	1	2	3	No	Yes	No	Yes	No	Yes
	**Relative Frequency (%)**															
	**Demographic Data**															
**Sex**																
F	**13.99**	**34.03**	**36.74**	**15.24**	**28.5**	**71.5**	**11.08**	**10.84**	**33.98**	**44.10**	14.4	85.6	77.25	22.75	49.8	50.2
M	**27.59**	**40.52**	**26.72**	**5.17**	**46.6**	**53.4**	**29.00**	**21.00**	**33.00**	**17.00**	13.0	87.0	83.50	16.50	57.8	42.2
**Age**																
<18	13.16	39.47	39.47	7.90	31.6	68.4	14.29	11.43	34.28	40	**5.3**	**94.7**	**87.5**	**12.5**	**35.1**	**64.9**
18-30	16.67	34.38	35.94	13.01	27.6	72.4	14.88	16.67	33.93	34.52	**7.9**	**92.1**	**84.9**	**15.1**	**52.8**	**47.2**
31-45	18.68	35.71	30.77	14.84	33.5	66.5	12.74	10.83	39.49	36.94	**14.3**	**85.7**	**80.9**	**19.1**	**50.0**	**50.0**
46-60	13.68	39.31	34.19	12.82	33.6	66.4	14.14	12.12	31.32	42.42	**22.3**	**77.7**	**73.7**	**26.3**	**61.5**	**38.5**
>60	18.18	27.27	40.91	13.64	37.9	62.1	19.64	8.93	21.43	50.00	**21.9**	**78.1**	**53.8**	**46.2**	**41.2**	**58.8**
	**Parafunctional Habits**															
**Clenching**																
No	15.72	42.14	33.96	8.18	34.6	65.4	**21.01**	**14.49**	**28.99**	**35.51**	14.7	85.3	80.2	19.8	51.8	48.2
Yes	13.37	35.38	38.16	13.09	30.4	69.6	**9.91**	**12.31**	**35.74**	**42.04**	13.7	86.3	78.4	21.6	48.9	51.1
**Bruxism**																

No	**14.62**	**44.34**	**31.60**	**9.44**	30.0	70.0	**16.58**	**16.06**	**30.57**	**36.79**	14.8	85.2	80.5	19.5	52.1	47.9
Yes	**12.78**	**32.22**	**39.44**	**15.56**	31.6	68.4	**7.14**	**10.12**	**33.33**	**49.41**	14.0	86.0	77.6	22.4	47.3	52.7
	**Risk Factors**															
**Dental treatment**																
No	16.90	34.58	35.95	12.57	33.1	66.9	14.61	12.33	34.02	39.04	14.4	85.6	79.3	20.7	49.7	50.3
Yes	15.12	39.53	27.91	17.44	25.6	74.4	14.29	15.58	32.47	37.66	12.8	87.2	73.7	26.3	61.0	39.0
**Orthodontic treatment**																
No	17.70	34.75	34.12	13.43	33.5	66.5	15.54	11.78	32.33	40.35	**15.9**	**84.1**	77.3	22.7	52.1	47.9
Yes	12.70	37.3	37.30	12.70	26.2	73.8	11.21	16.38	38.79	33.62	**8.0**	**92.0**	82.5	17.5	48.7	51.3
**Entubation**																
No	16.96	35.14	34.96	12.94	**32.7**	**67.3**	14.57	12.96	34.41	38.06	14.6	85.4	79.0	21.0	51.5	48.5
Yes	8.70	39.13	30.43	21.74	**13.0**	**87.0**	14.29	9.52	19.05	57.14	4.3	95.7	65.0	35.0	47.8	52.2
**Orthognatic Surgery**																
No	**16.70**	**35.26**	**35.26**	**12.78**	32.3	67.7	14.34	12.78	34.18	38.70	14.1	85.9	78.7	21.3	51.2	48.8
Yes	**12.50**	**37.50**	**0.00**	**50.00**	12.5	87.5	33.33	16.67	0.00	50.00	16.7	83.3	60.0	40.0	62.5	37.5
**Wisdom teeth removal**																
No	17.54	35.70	33.82	12.94	33.5	66.5	14.21	12.75	33.33	39.71	**15.7**	**84.3**	78.7	21.3	50.9	49.1
Yes	12.93	33.62	38.79	14.66	25.9	74.1	15.89	13.09	35.51	35.51	**8.0**	**92.0**	77.7	22.3	52.8	47.2
**Jaw Trauma**																
No	17.08	34.34	35.06	13.52	32.4	67.6	15.08	12.19	34.30	38.43	14.3	85.7	**79.4**	**20.6**	52.0	48.0
Yes	9.09	51.52	30.30	9.09	24.2	75.8	6.45	22.58	25.81	45.16	12.1	87.9	**63.3**	**36.7**	40.0	60
	**Other Comorbidities**															

**Number of other Comorbidities**																
0	**21.09**	**38.66**	**28.75**	**11.50**	32.1	67.9	**20.08**	**15.83**	**35.14**	**28.95**	12.1	87.9	78.9	21.1	52.7	47.3
1	**12.95**	**34.53**	**38.85**	**13.67**	36.0	64.0	**9.68**	**14.52**	**33.87**	**41.93**	13.0	87.0	81.1	18.9	50.4	49.6
≥2	**10.49**	**28.67**	**44.06**	**16.78**	28.0	72.0	**8.33**	**5.30**	**31.06**	**55.31**	19.3	80.7	75.0	25.0	49.2	50.8
**Mental, behavioral or neurodevelopmental disorders**																
No	**17,42**	**38.24**	**31.00**	**13.34**	31.5	68.5	**16.99**	**15.09**	**34.50**	**33.42**	13.8	86.2	78.0	22.0	51.9	48.1
Yes	**14.38**	**26.80**	**45.75**	**13.07**	33.3	66.7	**8.33**	**6.94**	**31.94**	**52.79**	15.0	85.0	79.7	20.3	49.6	50.4
**Diseases of the respiratory system**																
No	17.42	35.04	34.66	12.88	31.5	68.5	15.60	13.19	33.85	37.36	14.6	85.4	77.7	22.3	51.2	48.8
Yes	10.45	37.31	35.82	16.42	35.8	64.2	6.67	10.00	33.33	50.00	14.1	85.9	84.5	15.5	52.6	47.4
**Endocrine, nutritional or metabolic diseases**																
No	17.16	36.19	34.14	12.51	32.1	67.9	15.55	13.18	33.26	38.01	13.6	86.4	79.0	21.0	50.8	49.2
Yes	11.86	27.12	40.68	20.34	30.5	69.5	5.77	9.62	38.46	46.15	19.0	81.0	73.5	26.5	56.0	44.0
**Diseases of the circulatory system**																
No	16.58	35.17	34.99	13.26	31.7	68.3	14.71	13.01	33.69	38.59	**12.8**	**87.2**	79.5	20.5	51.9	48.1
Yes	17.31	36.54	32.69	13.46	34.6	65.4	13.04	10.87	34.78	41.31	**28.0**	**72.0**	67.4	32.6	45.5	54.5

**Diseases of the musculoskeletal system**																
No	17.17	35.60	34.53	12.70	31.7	68.3	14.40	13.17	33.95	38.48	14.1	85.9	79.1	20.9	50.7	49.3
Yes	8.33	30.56	38.89	22.22	36.1	63.9	17.24	6.90	31.03	44.83	14.3	85.7	66.7	33.3	61.3	38.7
